# Supplementary material for: Kinetics of Oil Absorption and Moisture Loss during Deep-Frying of Pork Skin with Different Thickness
Source: Foods. 2021 Dec 6;10(12):3029. doi: 10.3390/foods10123029 (PMC8702101; doi:10.3390/foods10123029)
Supplement: Supplementary file 1 [file foods-10-03029-s001.zip › foods-1458680-supplementary.pdf]

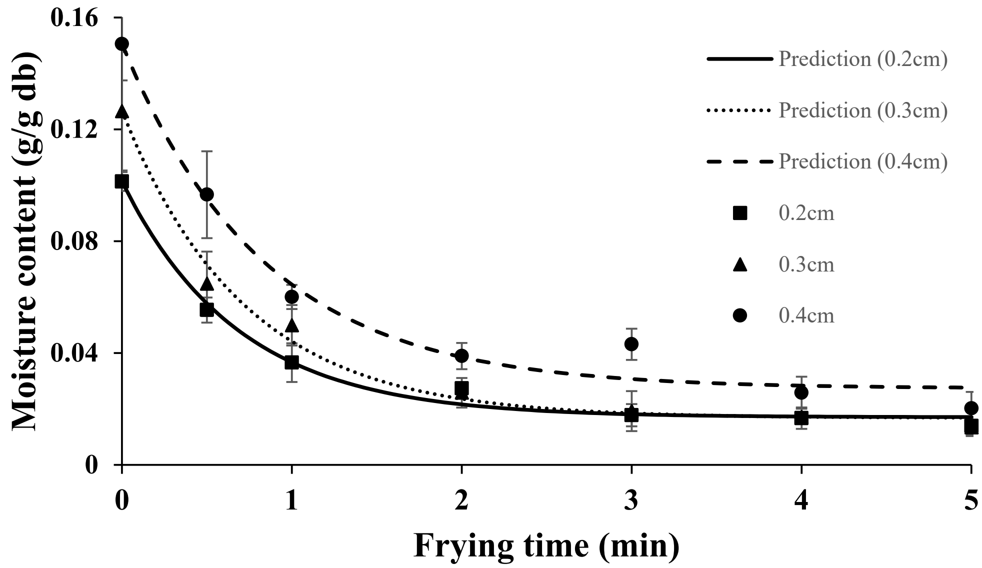

Figure S1. Moisture content of fried pork rind with different thickness and frying time. Experimental data and predicted by model equation (1).

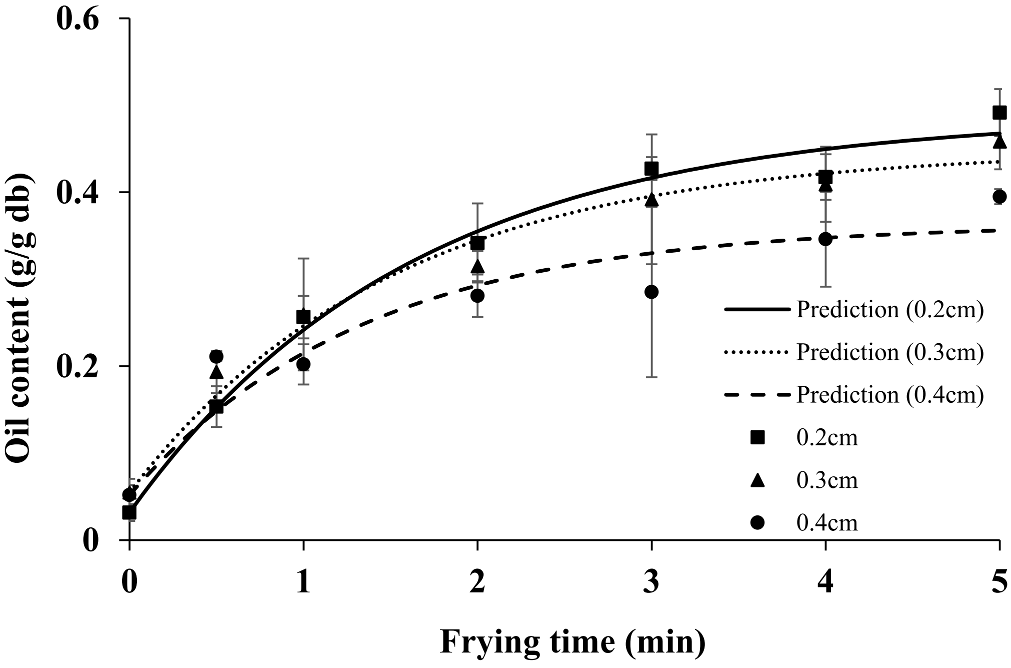

Figure S2. Oil content of fried pork rind with different thickness and frying time. Experimental data and predicted by model equation (2).

Table S1. The correlation coefficient between the frying time and overall properties of fried pork rind

**a**	**Frying time**	**Water content**	**Oil content**	**Breaking force**	**Water activity**	**The puffing ratio**	**Thickness after frying**
Frying time	1						
Water content	-0.796**	1					
Oil content	0.857**	-0.802**	1				
Breaking force	-0.506	0.757**	-0.571**	1			
Water activity	-0.790	0.943**	-0.770**	0.731**	1		
The puffing ratio	-0.008	-0.361**	0.006	-0.412**	-0.361	1	
Thickness after frying	0.378**	-0.129	0.134	0.067	-0.138	-0.180	1
**b**	**Frying time**	**Water content**	**Oil content**	**Breaking force**	**Water activity**	**The puffing ratio**	**Thickness after frying**
Frying time	1						
Water content	-0.803**	1					
Oil content	0.884**	-0.891**	1				
Breaking force	-0.645**	0.895**	-0.759**	1			
Water activity	-0.762**	0.951**	-0.810**	0.866**	1		
The puffing ratio	-0.008	-0.357**	0.006	-0.400**	-0.363**	1	
Thickness after frying	0.593**	-0.591**	0.566**	-0.458**	-0.502**	-0.180	1

** **indicate significance at** $p < 0.01$. (**a**) Not include t = 0 in statistical analysis (**b**) Include t = 0 in statistical analysis.

Table S2. The correlation coefficient between the thickness and overall properties of fried pork rind under the similar water content.

	**thickness**	**Water content**	**Oil content**	**Breaking force**	**Water activity**	**The puffing ratio**	**Thickness after frying**
thickness	1						
Water content	0.266	1					
Oil content	-0.519	-0.068	1				
Breaking force	0.858**	0.442	-0.446	1			
Water activity	-0.645	-0.273	0.348	-0.629	1		
The puffing ratio	-0.025	-0.288	-0.247	-0.247	0.214	1	
Thickness after frying	0.917**	0.013	-0.322	0.697*	-0.131	-0.402	1

** indicate significance at $p < 0.01$. * indicate significance at $p < 0.05$.

Table S3. The correlation coefficient of the results of fried pork rind's sensory evaluation.

	**Appearance**	**Smell**	**Flavor**	**Texture**	**Greasy Intensity**	**Overall Acceptability**
Appearance	1					
Smell	0.235**	1				
Flavor	0.205**	0.425**	1			
Texture	0.031	0.156*	0.472**	1		
Greasy Intensity	0.06	-0.061	-0.094	0.015	1	
Overall Acceptability	0.190**	0.314**	0.742**	0.666**	-0.048	1

** indicate significance at $p < 0$.
